# Taking ART to Scale: Determinants of the Cost and Cost-Effectiveness of Antiretroviral Therapy in 45 Clinical Sites in Zambia

**DOI:** 10.1371/journal.pone.0051993

**Published:** 2012-12-20

**Authors:** Elliot Marseille, Mark J. Giganti, Albert Mwango, Angela Chisembele-Taylor, Lloyd Mulenga, Mead Over, James G. Kahn, Jeffrey S. A. Stringer

**Affiliations:** 1 Health Strategies International, Oakland, California, United States of America; 2 Centre for Infectious Disease Research in Zambia, Lusaka, Zambia; 3 Zambian Ministry of Health, Lusaka, Zambia; 4 Center for Global Development, Washington, D.C., United States of America; 5 Super Models for Global Health, Oakland, California, United States of America; 6 Philip R. Lee Institute for Health Policy Studies, University of California San Francisco, San Francisco, California, United States of America; Indiana University, United States of America

## Abstract

**Background:**

We estimated the unit costs and cost-effectiveness of a government ART program in 45 sites in Zambia supported by the Centre for Infectious Disease Research Zambia (CIDRZ).

**Methods:**

We estimated per person-year costs at the facility level, and support costs incurred above the facility level and used multiple regression to estimate variation in these costs. To estimate ART effectiveness, we compared mortality in this Zambian population to that of a cohort of rural Ugandan HIV patients receiving co-trimoxazole (CTX) prophylaxis. We used micro-costing techniques to estimate incremental unit costs, and calculated cost-effectiveness ratios with a computer model which projected results to 10 years.

**Results:**

The program cost $69.7 million for 125,436 person-years of ART, or $556 per ART-year. Compared to CTX prophylaxis alone, the program averted 33.3 deaths or 244.5 disability adjusted life-years (DALYs) per 100 person-years of ART. In the base-case analysis, the net cost per DALY averted was $833 compared to CTX alone. More than two-thirds of the variation in average incremental total and on-site cost per patient-year of treatment is explained by eight determinants, including the complexity of the patient-case load, the degree of adherence among the patients, and institutional characteristics including, experience, scale, scope, setting and sector.

**Conclusions and Significance:**

The 45 sites exhibited substantial variation in unit costs and cost-effectiveness and are in the mid-range of cost-effectiveness when compared to other ART programs studied in southern Africa. Early treatment initiation, large scale, and hospital setting, are associated with statistically significantly lower costs, while others (rural location, private sector) are associated with shifting cost from on- to off-site. This study shows that ART programs can be significantly less costly or more cost-effective when they exploit economies of scale and scope, and initiate patients at higher CD4 counts.

## Introduction

Zambia is among the countries most severely affected by the HIV/AIDS epidemic. Prevalence among adults was between 14.3 and 16.4% in 2007. [1] Provision of free treatment started in April 2004, with support from the Global Fund to Fight AIDS, Tuberculosis and Malaria which in 2004 committed $254 million over 5 years; and from the President’s Emergency Fund for AIDS Relief (PEPFAR). Zambia is among PEPFAR’s most highly-funded countries, receiving $271.1 million in fiscal year 2009 and $276.7 in fiscal year 2010. [2] At the end of 2009, 68% of the 330,000 people in Zambia needing antiretroviral therapy (ART) were receiving it, and a third of all health facilities in the country were able to offer treatment. [3] As one of PEPFAR’S high priority “focus countries”. Zambia has made substantial progress toward universal treatment access.

While expansion of treatment services has proceeded rapidly, the available resources are now being strained by two very different changes. On the demand side, the bar was raised for what constitutes “Universal access” in November, 2009, when new World Health Organization (WHO) guidelines were released recommending an increase in the CD4 threshold for starting ART from <200 cells/uL to <350 cells/uL. This change, once adopted by countries, will instantly double the number of people eligible for therapy. This increasing demand for services occurs within a context in which the number of new infections exceeds the number of people placed on life-long ART each year by 2.5 to 1. [4] On the supply side, we are entering an era in which AIDS funding by major donors appears to be flattening. [5].

The Obama administration’s 2011 PEPFAR enacted budget totaled $6.8 billion, down from $6.9 billion in the previous year. [6]Now, more than ever, it is important to pay close attention to the costs and cost-effectiveness of ART in Africa. Such an understanding will help to ensure that available treatment dollars benefit as many people as possible and that the trade-offs between spending on HIV treatment and other global health needs are accurately quantified.

Evaluations of clinical outcomes of the Zambia ART program demonstrate that it is both feasible and successful. [7] In this article, we assess the cost and cost-effectiveness of the program for individual health centers and as a whole. Additionally, we examine the correlates of variation in unit-costs and cost-effectiveness across the 45 health centers.

## Methods

### Ethics Statement

The routine patient data reported in this analysis were deemed exempt from human subjects review by the Institutional Review Boards of the University of Zambia, the US Centers for Disease Control and Prevention, and the University of Alabama at Birmingham.

### Setting and Intervention

The Zambian government began offering free ART services in the public sector in early 2004, when the Centre for Infectious Disease Research in Zambia (CIDRZ) received PEPFAR funding from the US CDC to assist in scale-up. CIDRZ financial support to the health sector includes: (1) training in HIV clinical care, adherence support, pharmacy, data management, and clinic operations; (2) renovation and expansion of clinical and laboratory facilities; (3) pharmaceutical and laboratory reagent procurement; and (4) support for clinic staffing. Implementation of services was staged over time, with the order of roll-out determined by a given facility’s capacity to deliver services and by the underlying disease burden in the community it served. A detailed program description has been provided elsewhere. [8].

The present study selected for analysis all 45 ART centers in four Zambian provinces that CIDRZ had been supporting for at least 6 months at the time of data lock and analysis. Sites varied by setting, facility type, and primary funding source. These centers are located in four provinces, Thirty-eight of the 45 sites are located in urban areas; seven are classified as rural. Twenty-nine are clinics and 16 are hospitals. Six are private while 39 are predominantly publically-funded.

Clinical care at all sites is delivered according to standard national protocols. At time of enrollment, patients are screened for ART eligibility which is contingent on meeting one or more of the following criteria: (1) CD4 count <200 cells/uL, (2) WHO clinical stage 4 disease, or (3) CD4 count <350 cells/uL and WHO clinical stage 3 disease. Before July 2007, all eligible patients were initiated on a first-line ART regimen that included a nucleoside reverse transcriptase inhibitor (NRTI) backbone – lamivudine (3TC) plus either zidovudine (ZDV) or stavudine (d4T) – in combination with a non- nucleoside reverse transcriptase inhibitor (NNRTI); either efavirenz (EFV) or nevirapine (NVP). In July 2007, tenofovir disoproxil fumarate (TDF) plus either emtricitabine (FTC) or 3TC became the national standard first-line therapy.

Patients initiating ART have a more intensive visit schedule over the first 6 months to ensure they are able to adhere to their regimen and are not experiencing drug toxicities. Afterward, they are seen by a clinician every 3 months with a CD4 measurement recommended every 6 months. Viral load testing is not routinely available. The program tracks patients, their clinical care, and outcomes with a nation-wide electronic medical record system called “SmartCare.”

### Overview of Analytic Methods

We developed a computer-based, deterministic cost and cost-effectiveness model implemented in Microsoft Excel®. We distinguished costs at the facility (“on-site costs”) from the support costs incurred above the facility for monitoring, supervision, referral, training and administration of the CIDRZ ART program (“off-site costs”). Using regression analysis, we estimated the effect on average cost per person-year of treatment and its components of facility characteristics that may be determinants of those costs. We assessed health benefits consisting of averted deaths and associated averted disability-adjusted life-years (DALYs) from a health system perspective. Univariate sensitivity analyses were conducted to assess the effect of uncertainty. A 20,000-trial Monte Carlo simulation assessed the uncertainty from selected inputs (@Risk® version 5.7, Ithaca, NY). The cost per person-year of treatment and the cost per DALY averted are separate outcomes of interest.

Costs and health outcomes were used to estimate the incremental cost-effectiveness of ART added to an ongoing program of intended cotrimoxazole prophylaxis in the context of the national ART program implemented at 45 sites. Program costs and outputs were tabulated from services initiation on April, 26 2004 through July 1, 2008 when the data set was declared closed. We report facility-level unit costs as the full incremental cost of providing ART divided by the number of person-years on treatment. Cost-effectiveness is reported as program cost per DALY averted and per death averted. [9] Age-weights were excluded in calculating DALYs. [10] We standardized our model calculations to 100 person-years of ART and discounted future costs and benefits at a rate of 3% per year. [9] Costs and benefits were modeled 10 years into the future.

The following facility-level variables were selected *a priori* as possible predictors of program efficiency: setting (urban versus rural); scope of the facility’s output (i.e. hospital versus clinic); predominate funding source (public versus private); program scale measured as the number of person-years of ART provided per year of site experience; portion that pediatric ART-years constitutes of all ART-years provided; median patient age, median CD4 cell count at treatment initiation; proportion of patients with WHO disease stage 4 at treatment initiation; years of program experience; and adherence measured as a variation of the commonly-reported medication possession ratio (MPR). [11], [12].

### Cost of ART Program

We conducted a comprehensive accounting of the incremental resources consumed by ART-related activities and their costs, applying standard micro-costing methods in which each type of resource consumed, such as medications, lab tests and personnel time, is quantified and assigned a unit cost. [13] We excluded the cost of the initial HIV test and of pre-ART monitoring. Historical salary and expenditure records were queried from AccPac (Irvine, California, USA), the comprehensive electronic accounting system used to manage finances for the CIDRZ program. We performed additional queries to quantify expenditures on compensation for government clinical and support personnel required by the ART program at each site and for both administrative and direct services support provided by CIDRZ staff in Lusaka. Expenditures for community outreach, and peer education were derived from CIDRZ program records of expenditures for contracted services and for outreach coordination performed by CIDRZ staff. These costing data were verified with interviews of project managers, service delivery staff, and accounting staff (Table 1).

**Table 1:** CIDRZ ART services delivered and associated costs in 45 clinical sites in Zambia.

		Range across 45 sites			
	Totals: all sites	50th Percentile (Inter-Quartile Range)	Standard deviation	Minimum	Maximum
**Services delivered**					
Number of adults starting ART	84,671	1,177 (652–2,889)	1,609	111	6,622
Number of children starting ART	5,785	82 (33–158)	126	4	471
Total starting ART	90,456	1,266 (700–3,243)	1,723	116	7,093
Adult-years on ART	117,234	1,464 (524–4,263)	2,653	78	10,910
Child-years on ART	8,202	90 (30–203)	214	3.0	806
Total person-years on ART	125,436	1,535 (559–4,689)	2,853	81.0	11,716
Years of ART service provision	117	2.6 (399–1,288)	1.02	1.0	4.2
Average PYs of treatment per year per site	886	700 (399–1,288)	693	55.5	2,804
Average years on ART per adult	1.38	1.2 (0.9–1.5)	0.37	0.6	1.8
Average years on ART per child	1.42	1.1 (0.9–1.4)	0.38	0.5	2.0
**Program costs**					
**Personnel** 1	**$16,702,251**	**$203,725 ($73,315−$574,728)**	381,636	**$8,743**	**$1,607,807**
Salary and program support	$14,305,132	$169,938 ($65,465–$508,774)	$320,886	$7,778	$1,336,434
Peer Educators and Community outreach	$2,397,119	$25,903 ($12,522–$65,954)	$62,307	$966	$271,374
**Recurring goods and services**	**$50,949,372**	**$601,357 ($270,408−$1,633,158)**	**$1,118,781**	**$48,960**	**$4,267,653**
ARV drugs; essential medications; misc medical supplies	$31,040,710	$381,830 ($141,472–$1,149,522)	$715,281	$15,613	$2,583,227
Administrative support services and supplies	$10,828,269	$147,836 ($98,056–$344,325)	$188,690	$25,683	$750,970
Laboratory costs	$9,080,393	$96,274 ($24,785–$258,605)	$243,672	$7,664	$952,070
**Training**	**$1,543,447**	**$18,822 ($6,136−$58,486)**	**$35,785**	**$711**	**$149,198**
**Facilities** 2	**$506,274**	**$4,587 ($2,434−$11,544)**	**$17,198**	**$831**	**$93,003**
***Total***	***$69,701,346***	***$814,716 ($398,519*** **−** ***$2,412,623)***	***$1,540,752***	***$60,206***	***$6,049,736***
**Cost of ART per year**	**$556**	**$569 ($499−$726)**	**$205**	**$413**	**$1,237**
**Epidemiological inputs**					
Percent female	59.9%	60.4% (58.6%−62.0%)	3.8%	42.6%	67.0%
Percent adults	93.8%	93.6% (92.6%−95.7%)	2.5%	87.3%	98.9%
CTX status	60.7%	52.1% (45.9%−81.6%)	22.7%	4.5%	95.9%
Percent with WHO stage 4	11.7%	10.5% (8.2%−15.2%)	5.4%	4.5%	26.3%
**Deaths per 100 PY in CIDRZ (45 sites)** 3					
Baseline CD4∶000–050	4.6	4.2 (1.5–6.6)	7.2	0.0	40.8
Baseline CD4∶050–199	2.9	2.7 (0.7–3.7)	1.8	0.0	7.2
Baseline CD4∶200+	2.4	2.4 (0.0–3.4)	5.7	0.0	37.6
**Deaths per 100 PY in HBAC comparison group**					
Baseline CD4∶000−050	116	NA			
Baseline CD4∶050−199	27	NA			
Baseline CD4∶200+	7	NA			
**Rate of annual switch from first to second-line ARV regimen**					
Year 1	0.8%	NA			
Year 2	1.1%	NA			
Year 3	2.1%	NA			
Year 4	3.0%	NA			
Year 5	3.4%	NA			
Discounted DALYS averted per death averted	7.3	NA			
**Discount rate**	0.03	NA			

1 Excluding training and laboratory.

2 Refurbishment - renovation and imputed opportunity cost of space.

3 Adjusted for difference in % female clients between HBAC and CIDRZ cohorts.

Program phased in April 28, 2004–July, 15, 2007; data through July 1, 2008. All values derived from CIDRZ program records except Hazard ratio: Female - male and Deaths per 100 PY in HBAC comparison group (Mermin, 2008) and Discounted DALYS averted per death averted (Marseille, 2009). Annual regimen switch rates, death rates in the comparison group, DALYs per death averted and the discount rate are constant across sites but are varied from 50% to 150% in the sensitivity analyses.

Expenditures for medical supplies, miscellaneous office supplies and drugs for the treatment of opportunistic infections, were obtained from the electronic medical record system that provides the quantity and unit cost of all items dispensed at each site. To calculate the cost of antiretroviral drugs we totaled the number of each type of first and second-line dispensation given at each site and adjusted these by the adherence rate. We then multiplied the adjusted number of each dispensation by the cost of the respective regimen. The cost of the regimen, in turn, is what CIDRZ paid for the drugs composing that regimen. Data to adjust these costs by potential wastage were unavailable, and we did not adjust for possible waste. “Administrative support services, supplies and equipment” consisted of support for monitoring and supervision, and supplies and equipment for headquarter support operations such as data management and IT, telecommunications, vehicle maintenance, legal and insurance costs, utilities, and other miscellaneous services. We assigned these central administrative support expenditures to each site in proportion to the number of ART service-years provided by each site in each respective year. In cases in which a line-item was designated as support for the site within a particular province only, this sum was allocated to each clinical site within the province in proportion to the person-years of ART delivered for each year and site within that province.

The frequency of laboratory testing for a given patient was derived from the SmartCare electronic medical record. The cost of each laboratory test was derived from the “re-charge” model in use at the CIDRZ central laboratory in Lusaka. This model, which is used to apportion laboratory costs to various projects according to their real resource consumption volume, considers the cost of reagents, expendable supplies, equipment depreciation, and incremental labor used for each test.

Since most training courses were provided at the Province or District level (i.e., to multiple sites simultaneously), we allocated the cost of each training session to each participating site according to its proportion of ART person-years among the trained group. To quantify facility costs we calculated the opportunity cost of building space by multiplying the area dedicated to ART at each site by an average monthly rental cost per square meter of $3.20 per square meter. All costs were converted from Kwacha to US dollars at the exchange rate available on June 30 of the relevant year (http://www.xe.com/currencycharts/?from=USD&to=ZMK&view=10Y). They we then adjusted to 2010 US dollars according to the consumer price index.

Table 2 presents the allocation of costs between on-site components constituting two-thirds of all costs and the balance to off-site costs. Off-site costs include expatriate personnel ($4.0 million), off-site goods and supplies ($10.4 million) central (as opposed to on-site) training ($0.3 million) and the cost of buildings ($0.5 million).

**Table 2:** Distribution of cost sub-components between total and on-site components.

	Cost items	Total (All sites)	Intermediate cost aggregates	Totals	Cost aggregatesas % of Total insub-category	On-site Totals	Cost components as % of Total in sub-category
**Personnel cost**	Salary support (“Shift”) costs	$1,515,219	Personnel On-Site	$4,473,999	26.27%	$4,473,999	100.00%
	DHMT salary support and ARV nurses	$561,661					
	Peer Educators	$208,822					
	Outreach semi-volunteers (follow up)	$693,135					
	Community Outreach via PCI and CIDRZ	$1,495,162					
	Lusaka-based personnel costs - Zambian	$8,225,473	Personnel Off-site	$12,228,252	73.2%		
	Lusaka-based personnel costs - Expatriate	$4,002,779					
	**Personnel Sub-total**	**$16,702,251**	**Personnel Sub-total**	**$16,702,251**	**24.0%**	**$4,473,999**	**9.6%**
**Recurring goods and services**	ARV drugs (CIDRZ Accounts)	$29,549,541	ARV drugs	$29,473,910	57.8%	$29,549,541	72.9%
	Other essential drugs	$1,192,486	Goods On-site	$1,898,750	3.7%	$1,898,750	4.7%
	Medical supplies	$298,683					
	Office supplies	$407,580					
	Laboratory costs by site	$8,721,121	Lab costs	$9,080,393	17.8%	$9,080,393	22.4%
	Non-site specific lab costs	$359,272					
	Province/site-specific Lusaka-based support	$3,423,121	Goods Off-site	$10,420,689	20.5%		
	Non-province/site-specific Lusaka-based support	$6,997,568					
	**Goods and service Sub-total**	**$50,949,372**	**Goods and service** **sub-total**	**$50,949,372**	**73.1%**	**$40,528,684**	**86.6%**
**Training**	Non-province-specific	$264,171	Training Central	$264,171	17.1%		
	Lusaka	$940,643	Training site	$1,279,276	82.9%	$1,279,276	100.00%
	Southern	$165,155					
	Western	$86,804					
	Eastern	$86,675					
	**Training Sub-total**	**$1,543,447**	**Training Sub-total**	**$1,543,447**	**2.2%**	**$1,279,276**	**2.7%**
**Building space**	Buildings (opportunity cost of space)	$60,879	Buildings Sub-total	$506,274	n/a	$506,274	100.00%
	Renovations for specific provinces (amortized)	$13,472					
	Renovations for unspecified sites (amortized)	$204,795					
	Renovations for specified sites (amortized)	$227,129					
	**Buildings Sub-total**	**$506,274**	**Buildings Sub-total**	**$506,274**	**0.7%**	**$506,274**	**1.1%**
			**Total program costs**	**$69,701,345**	100.00%	**$46,788,234**	100.00%

### Health Effects of ART Program

The electronic medical record system does not track a comparable control population of patients who are eligible for, but not receiving ART. We therefore elected to compare the on-ART survival of our cohort with a well-characterized home-based AIDS care (HBAC) cohort of 466 HIV-infected patients followed in Tororo District, Uganda. [14] Beginning in April 2001, this group was followed for two years; patients were given no intervention during the first 5 months and then provided with daily cotrimoxazole prophylaxis (CTX) for an additional 1.5 years. The program benefit is the lower number of deaths observed in the CIDRZ facilities compared with what would have occurred without treatment. Deaths without treatment were the empirical observations of the HBAC control (CTX-only) groups within each of three CD4 strata, (0–50; 51–200 and 200+). To standardize the varying lengths of time different sites provided ART, mortality reduction is expressed as the number of averted deaths per 100 years of ART. Deaths per 100 years of ART in the HBAC cohort were 116, 27 and 7 for 0–50, 51–199 and 200+ CD4+ strata respectively at ART initiation. For the CIDRZ cohort, the corresponding deaths per 100 person-years of ART were 4.6, 2.8 and 2.4. (Table 1). The number of deaths averted at each CIDRZ-supported site thus depends on the observed death rate in each CD4 stratum at that site, and the proportion of clients in each stratum. Women comprised 59.9% of the CIDRZ study cohort versus 75.0% of the HBAC cohort. There was an excess mortality observed in HBAC males compared with males found in the CIDRZ ART sites (hazard ratio 1.4,). Applying this hazard ratio, the CIDRZ death rate estimates were adjusted for differences in the male: female ratio at each CIDRZ site compared with the HBAC cohort.

Mortality rates tend to fall dramatically following 3–4 months of ART treatment compared with the prior period. [8]. We therefore represent results two ways, limiting the analysis to events occurring after 16 weeks when the benefits of ART start to be clinically significant, and also including the entire time of treatment.

### ART Program Cost-Effectiveness

We calculated cost-effectiveness using six different metrics. We considered ART’s effect on mortality starting after 16 weeks. [14] We also considered mortality rates for the cohort from the start of ART. We then applied three different measures of the cost of ART for the additional years of life resulting from treatment. The first method applies an estimate of the future cost of ART based on the average discounted cost per person-year for the entire program. This method tacitly assumes that the variations in cost by site converge into a uniform unit cost for the whole program. The second method uses the observed cost of ART at each site for purposes of estimating future costs. This method thus preserves the by-site variation in costs as these are projected into the future. The site-specific estimates also accurately reflect the distribution of antiretroviral regimen types and their associated costs at each site and assume that the differences in ART costs by site will be preserved. For both these methods, discounted future costs reflect the switch rate from first to second line therapy observed for the whole program. This was 0.77%, 1.06%, 2.13%, 3.00 and 3.43 percent for years 1 through 5 of treatment respectively. This is an average annual increase in the switch rate of 34.9%. For the remaining 5 years of our projection, we assume a constant switch rate increase of 34.9%. We estimated that 7.3 discounted DALYs are averted per AIDS death averted. This figure was obtained by eliminating age weights from the 7.6 discounted future DALYS calculated for the HBAC cohort in Uganda. [15] The third approach to treating future cost and benefits is to confine the analysis to the period of empirical observation, with no projection of future costs or benefits.

## Results

Between April 26, 2004 and July 1, 2008, the 45 health centers examined in this study provided a median of 2.6 years of service (Standard Deviation [SD]: 1.02). Incremental ART expenditures were $70.4 million for 125,436 person-years of ART delivered. Of the 90,456 patients who received ART, 5,785 were children under five. The median site provided 1,535 person-years of ART during the study period (SD: 2,853).

### ART Program Unit Costs

**Figure 1:**
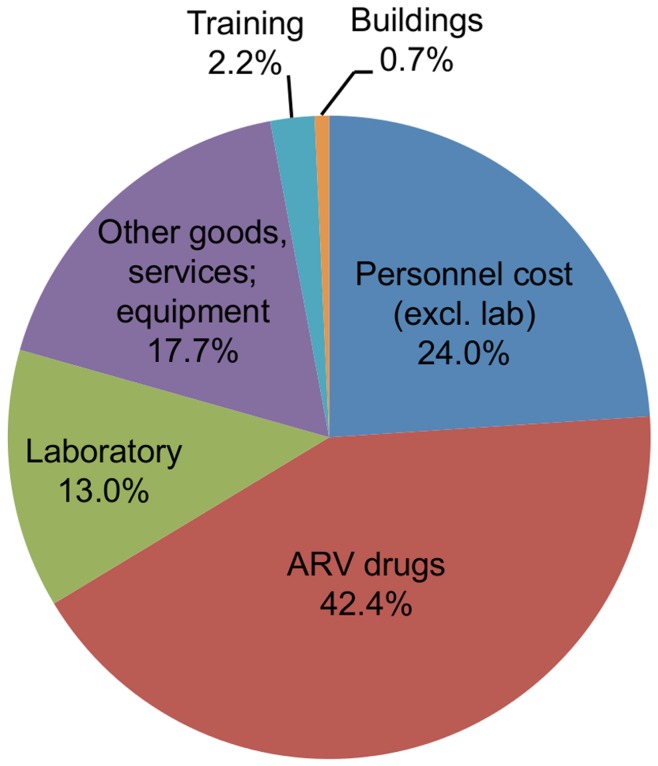
Breakdown of incremental ART program costs by major expenditure categories.

The incremental cost of the program considered as whole (obtained by summing the incremental costs of all 45 sites and dividing by the person-years of service provided) was $556 per person-year of ART. Antiretroviral drugs were the single largest cost (42.4% of total, SD: 8.3%) followed by personnel (24.0%, SD: 3.8%); non-medication goods and services and amortized equipment (17.7%, SD: 11.0%); and laboratory costs (13.0%, SD: 5.6%). (Figure 1.) The average facility incurred total costs of $638 per person-year of treatment, of which $428 were on-site costs and the balance off-site.

### Cost-effectiveness

Considering only mortality benefits following 16 weeks of ART and using site-specific program costs to project the future cost of ART for averted deaths, the program cost $833 per DALY averted (SD: $316) or $6,118 per death averted (SD: $2,323). Using average program costs to estimate future ART cost, yields $898 (SD: $160) and $6,601 (SD: $1,179) per DALY and death averted, respectively. Finally, considering only the results observed within the study period, and projecting no future costs or benefits, yields $1,210 (SD: $2,262) and $1,668 (SD: $590) per DALY averted and per death averted, respectively. (Table 3).

**Table 3:** CIDRZ Zambia ART program cost-effectiveness in 45 sites.

	Costs per DALY averted(Mean and standard deviation)		Cost per death averted(Mean and standard deviation)	
	Benefits calculated after 16 weeks of ART	Benefits calculated from start of ART	Benefits calculated after 16 weeks of ART	Benefits calculated from start of ART
Cost of future ART assuming future costsper PY based on observed variation incosts by site.	$833 ($316)	$1,065 ($678)	$6,118 ($2,323)	$7,822 ($4,985)
Cost of future ART assuming future costsper PY are the same as the currentpooled average for all sites	$898 ($160)	$1,149 ($496)	$6,601 ($1,179)	$8,440 ($3,646)
No projection of future costs or benefits; results confined to empirical data	$1,210 ($2,262)	$1,550 ($3,935)	$1,668 ($590)	$2,133 ($1,172)

Cost effectiveness is presented as costs and mortality reductions following initial 16 weeks of therapy; and for the entire period between ART services initiation at each site and July 1, 2008. Means are the sum of all DALYs or deaths averted across all sites, divided by the sum of the costs at all sites. Standard deviations are calculated for the mean values of all sites.

### Predictors of Efficiency and Cost Effectiveness

We analyzed the associations between eight predictors of average cost and cost-effectiveness and our measures of efficiency and cost-effectiveness. We present simple linear models, but find that our explanatory power improves when we use the less skewed logarithmic transformation of the dependent variables and when we map all the predictors into dummy variables indicating whether a facility is above or below a critical threshold.

#### Bivariate analysis

Three of the rows of Table 4 reveal statistically significant correlations with all three cost categories. Hospitals are associated with lower on-site and off-site costs, and therefore with lower average total cost (p<.01 for on-site and total and p<.05 for average off-site cost). Months of ART experience is a continuous variable with a mean of 31.2 months in the sample, which we have mapped into a dichotomous variable equal to one if the facility has more than 24 months experience. The continuous version of months of experience (row e) is negatively related to the total and the off-site average costs, while the dummy variable is negatively associated with all three cost measures (p<.01). The continuous measure of scale of production (row f) is associated with lower off-site and total costs and its association with lower on-site costs has a p-value of 0.11.

**Table 4:** Bivariate analysis of association between average total cost and its components and eight explanatory variables.

					Three dependent variables measuring the efficiency of ART service delivery											
	Explanatory variables				Average facility total cost per person-year ($645)				Average facility on-site cost per person-year ($394)				Average facility off-site cost per person-year ($251)			
	(1)	(2)	(3)	(4)	(5)	(6)	(7)	(8)	(9)	(10)	(11)	(12)	(13)	(14)	(15)	(16)
	Variable name	Dummy definition	Mean	SD	Correlation	For D = 0	For D = 1	p-value	Correlation	For D = 0	For D = 1	p-value	Correlation	For D = 0	For D = 1	p-value
a)	**Adherence**	continuous	0.90	0.06	0.316	.	.	0.034	0.406	.	.	0.006	0.129	.	.	0.397
		1 = adhr>91%	0.64	0.48	.	558.2	682.3	0.050	.	319.1	429.2	0.001	.	239.1	253.1	0.747
b)	**Baseline CD4**	continuous	145.19	24.19	−0.133	.	.	0.383	−0.073	.	.	0.635	−0.137	.	.	0.369
		1 = CD4>144	0.40	0.50	.	661.9	602.6	0.347	.	403.1	370.6	0.362	.	258.8	232.0	0.528
c)	**Who stage 4**	continuous	0.12	0.05	0.141	.	.	0.354	0.060	.	.	0.698	0.160	.	.	0.292
		1 = Stage<10%	0.47	0.51	.	673.5	597.8	0.220	.	407.7	369.9	0.280	.	265.8	227.9	0.362
d)	**Hospital vs Clinic**	1 = Hospital	0.36	0.48	.	721.8	486.7	<0.001	.	439.9	299.9	<0.001	.	281.9	186.8	0.025
e)	**Months since start of ART delivery**	continuous	31.16	12.22	−0.386	.	.	0.009	−0.143	.	.	0.349	−0.454	.	.	0.002
		1 = >24	0.64	0.48	.	787.4	555.9	<0.001	.	442.1	361.4	0.023	.	345.3	194.5	<0.001
f)	**Patient-years per year**	continuous	886.13	692.93	−0.505	.	.	<0.001	−0.225	.	.	0.138	−0.563	.	.	<0.001
	** Dpyr300**	1 = >300	0.78	0.42	.	899.3	563.6	<0.001	.	460.2	370.1	0.028	.	439.1	193.5	<0.001
	** Dpyr800**	1 = >800	0.44	0.50	.	719.7	536.4	0.002	.	412.1	362.5	0.155	.	307.5	173.8	<0.001
g)	**Rural vs urban**	1 = Rural	0.16	0.37	.	612.4	778.0	0.048	.	391.0	385.2	0.905	.	221.5	392.8	0.002
h)	**Private vs public**	1 = Private	0.13	0.34	.	632.6	674.6	0.645	.	393.1	370.4	0.660	.	239.5	304.2	0.288

Column definitions:

(1) Variable definitions.

(2) Specifies whether variable is continuous or discrete, and if discrete, the criterion used to define the dummy variable.

(3) Mean value of the explanatory variable over all 45 observations. For discrete versions of the variables, the mean is the proportion of observations for which D  = 1.

(4) Standard deviation of the explanatory variables.

(5) Correlation between a continuous explanatory variable and average total cost.

(6) Mean value of average total cost for values of the dummy variable equal to zero.

(7) Mean value of average total cost for values of the dummy variable equal to one.

(8) p-value of the test of the null hypothesis of no relationship between the explanatory variable and average total cost.

(9) – (12): Same definitions as (5) – (8), respectively, except for average on-site cost instead of average total cost.

(13) – (16): Same definitions as (5) – (8), respectively, except for average off-site cost instead of average total cost.

Facilities that achieve higher adherence rates have higher on-site costs (correlation coefficient 0.4 with p-value.006) and a dummy variable defined at a threshold of 0.91 is also statistically significant and associated with about $110 dollars of additional on-site costs. Rural facilities are associated with higher off-site costs (p-value.002). In this bivariate analysis, measures of the complexity of admitted patients (baseline CD4 and WHO Stage at admission) are not statistically significant and whether the facility is in the public or private sector seems unrelated to any cost component.

#### Multivariate analysis

Table 5 presents multivariate regression analysis of average total cost and its components, on-site and off-site cost as explained by the same variables included in the bivariate analysis above. Columns (1) through (3) present linear regressions. Because the dependent variable in column (1), average total cost, is defined as the sum of the dependent variables in columns (2) and (3), the coefficient in column (1) for any explanatory variable is identically equal by construction to the sum of the coefficients in columns (2) and (3) for the same variable. A coefficient can be interpreted as the estimated increment to average cost associated with a one-unit increase in the given explanatory variable. Some independent variables have a statistically significant estimated effect on one component of average cost, some on both and some only on the total. The last two variables, dummies for rural and private sector, have opposite statistically significant effects on on-site and off-site costs, such that their estimated impacts on average total cost offset one another and are therefore statistically insignificant in that regression.

**Table 5:** Estimated regressions of average total and average on-site cost per patient-year of antiretroviral therapy in 45 facilities in Zambia.

	Individually estimated linear regressions			Criterion for defining dummy variable	Jointly estimated regressions of logged dependent variable on dummy variables∧^a^		Point estimates and 95% confidence intervals for predicted average cost per patient∧^b^	
Column numbers	(1)	(2)	(3)	(4)	(5)	(6)	(7)	(8)
Dependent variables	Averagetotal cost	Averageon-site cost	Averageoff-site cost	NA	Natural log of average total cost	Natural log of average on-site cost	Average total cost	Average on-site cost
**Explanatory variables**								
Constant	951.66** (0.050)	395.99 (0.255)	555.67 (0.200)	Constant	6.824**(0.000)	6.208**(0.000)	919.8 (787.6–1,074.1)	496.6 (412.5–597.8)
Proportion of patients adherent	248.02 (0.545)	256.83 (0.433)	−8.82 (0.973)	>.91	0.104**(0.067)	0.156**(0.021)	1,020.3 (894.3–1,164.1)	580.3 (495.6–679.3)
Average baseline CD4	−1.77+ (0.132)	−0.60 (0.336)	−1.16 (0.229)	>144	−0.128**(0.008)	−0.108*(0.061)	859.5 (741.8–995.9)	470.1 (394.2–560.7)
Proportion of patients initiatingat WHO Stage 4	711.34* (0.090)	256.83 (0.397)	454.76 (0.251)	<.10	−0.044 (0.361)	−0.103*(0.071)	686.3 (574.8–819.5)	352.4 (285.1–435.6)
Dummy = 1 if Hospital	−241.05** (0.000)	−152.35** (0.000)	−88.70 (0.004)	= 1 if Hospital	−0.225**(0.000)	−0.288**(0.000)	575.1 (461.1–717.3)	297.2 (228.2–387.0)
Months of ART service provision	−7.32** (0.004)	−2.46 (0.200)	−4.86** (0.023)	>24	−0.177**(0.005)	−0.170**(0.022)	468.8 (403.4–544.7)	300.3 (250.9–359.3)
Patient-years of ART per yr of operation	−0.05* (0.075)	−0.03+ (0.198)	−0.03 (0.276)	>300	−0.204**(0.008)	0.010 (0.910)	423.0 (370.5–483.0)	276.0 (235.5–323.4)
				>800	−0.103**(0.07)	−0.084 (0.21)		
Dummy = 1 if Rural	−42.45 (0.657)	−111.73* (0.050)	69.28 (0.320)	= 1 if Rural	−0.035 (0.626)	−0.185** (0.033)	408.3 (332.9–500.9)	229.4 (179.7–292.9)
Dummy = 1 if Private	26.33 (0.594)	−65.98** (0.040)	92.31** (0.005)	= 1 if Private	0.007 (0.919)	−0.133+ (0.131)	411.4 (312.3–542.0)	200.8 (144.4–279.2)
Number of observations	45	45	45		45	45		
R-squared	0.636	0.539	0.556		0.782	0.659		
p of Early Start∧^c^	0.093	0.498	0.057		0.043			
p of Economies of Scale	0.084	0.206	0.283		0.000			

Note:**p<0.05,

* p<0.1,+p<0.2. p-values are given in parentheses below the coefficients in columns (1) through (6).

a Coefficients in columns (5) and (6) are estimated by Zellner’s seemingly unrelated regression method using Stata’s sureg command.

b Predicted values in columns (7) and (8) are calculated from the coefficients in columns (5) and (6) respectively, as explained in the text.

c Values of p for “Early Start” are the significance level at which the joint hypothesis can be rejected that neither baseline CD4 nor proportion of patients initiating at WHO stage IV influences the dependent variable. For the two equations estimated jointly (columns (5) and (6)) the p-value is the probability that these four estimated coefficients could occur if all four parameters were actually zero.

Columns (5) and (6) present estimates of our preferred specification to explain average total cost and average on-site cost. In order to test for the statistical significance of explanatory variables on all components of average cost, we estimate the cost functions jointly using the method of “seemingly unrelated regressions” as implemented by the *sureg* command in Stata 12. We dropped the average off-site cost from this joint estimation because its results are implied by the estimates of the other two equations. Average total cost and its components, like most health expenditure variables, have skewed distributions with long right tails. To avoid the potential for unusually influential outliers dominating the regression results, and to more consistently estimate the impacts of the explanatory variables on cost, we therefore used the logarithms of average total cost and on-site cost as the dependent variables in columns (5) and (6). We also replaced all of the continuous explanatory variables with dummy variables designed to detect a threshold of each above which average costs are different to a statistically significant degree. The definition of these dummy variables is given in column (4).

**Figure 2:**
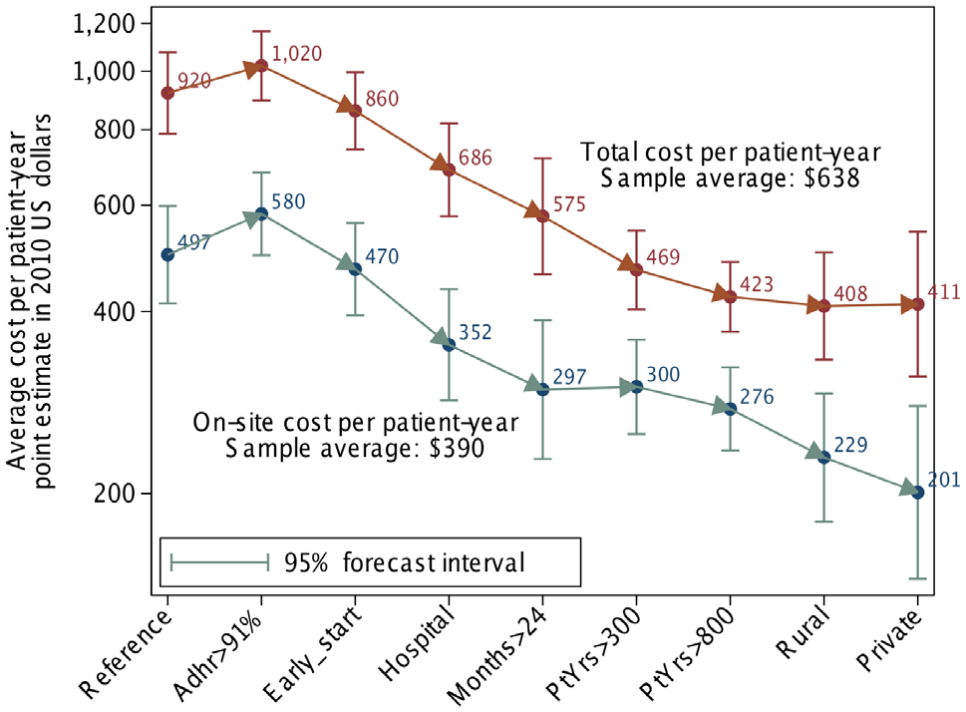
Estimated costs of ART service delivery per patient year by ART facility characteristic. Figure shows total and on-site costs. Author’s estimates constructed from the semilog specification in Table 5. The “reference” facility is an urban public sector clinic with less than 91% adherence, less than 2 years’ experience, fewer than 300 patients, and late starting patients. Estimated effects of facility characteristics accumulate from left to right.

Columns (7) and (8) of Table 5 apply the coefficient estimates from columns (5) and (6) to give point estimates and 95% confidence intervals for average total and on-site cost of ART. For these computations illustrated in Figure 2, the dummy variables are first all set to zero in the first row of the table and then sequentially switched to one down. The constant term in column (7) of $922 thus gives the estimated average total cost for the reference facility, an urban, public sector clinic with less than 24 months of experience fewer than 300 patient-years of annual output, late patient initiation (as captured by a low CD4 count or classification as WHO Stage 4 at initiation) and relatively poor adherence. In this same type of facility, we estimate average on-site cost to be $499. Estimated off-site cost is the difference between these two, or $423. Thus for this canonical facility, estimated on-site cost represents about 54% of average total costs per patient.

While the two measures of case complexity at initiation, the facility’s average baseline CD4 and the proportion of its patients initiated at WHO Stage 4, were not statistically significant individually in the linear specification, three of the four coefficients for these variables in columns (5) and (6) are statistically significant and the joint test for all four of these coefficients across the two equations has a p value of 0.015 (in the row labeled “p Early Start” near the bottom of the table). In columns (7) and (8), we model these two variables as jointly characterizing a facility that starts its patients earlier and estimate that adding these characteristics to a facility with good adherence, reduces the average total cost per patient-year by $147 down to $878. More than two thirds of the reduction in overall costs occurs at the facility, where costs decline to $482, while about 30% of the reduction occurs in the off-site costs.

The next four rows of columns (7) and (8) present estimates of the reductions in estimated average and on-site cost per patient-year that could be achieved if the facility were a hospital instead of a clinic, had more than 24 months experience, operated at a scale greater than 300 patient-years of service per year, and then greater than 800 patient-years per year. These changes jointly bring the average total cost per patient-year down to $432 dollars per patient-year of which $282, or almost two-thirds, is incurred on-site. The coefficients in columns (5) and (6) from which we derive these effects are all either individually or jointly significant. The four coefficients capturing scale effects are jointly significant with a p-value less than 0.001.

Neither a rural setting nor private sector ownership is estimated to have a statistically significant effect on average total cost per patient-year. But both of these characteristics have an important effect on the share of total cost incurred at the facility as opposed to off-site. A facility that is otherwise identical to those modeled in the above row, reduces its on-site cost per patient year to $235 if it is in a rural setting and to $207 if it is in the private sector. But these cost reductions are off-set by an increasing cost burden above the facility level. Other things equal, a facility that is either rural or private bears an incremental burden of $33 per patient-year on the support structure above it. The linear specification in column (3) suggests that these burdens on the support system could be even higher.

The multivariate analysis of this observational data reveals remarkable heterogeneity in average total cost and its distribution between on- and off-site components across the 45 CIDRZ sites and succeeds in explaining 78% and 66% of the variation of average total and on-site costs respectively.

Table 6 presents our analysis of the variation in the average total cost per DALY averted using the explanatory variables and specifications of Table 5. The estimated coefficient of the proportion of patients adhering is larger in column (1) of Table 6, perhaps reflecting the greater contribution of good adherence to cost-effectiveness than to cost per se, but the coefficient is not statistically significant in either linear model. Of the two variables capturing early start, only the proportion initiating at WHO Stage 4 is statistically significant, but it’s coefficient is larger than in Table 5, suggesting that late initiation increases the cost per DALY even more than it increases the cost per patient year. According to column (1) every percentage point increase in the proportion of patients initiating increases the cost per DALY by about $14. Other coefficients suggest that, other things equal, “hospitals” and “more than 24 months of experience” both improve the efficiency of DALY production. While measures of scale do not have statistically significant coefficients in columns (1) and (2), they are highly significant at reducing costs in the dummy variable specification in column (4).

**Table 6:** Estimated regressions of average total cost per DALY averted of antiretroviral therapy in 45 facilities in Zambia.

Column numbers:	(1)	(2)	(3)	(4)
Dependent variables:	Average totalcost per DALYaverted	Natural logarithmof average total costper DALY averted	Criterion fordefining dummyvariable	Natural logarithm ofaverage total cost perDALY averted
**Explanatory variables**				
Constant	927.969 (0.277)	6.669** (0.000)	Constant	7.216** (0.000)
Proportion of patients adherent	509.005 (0.490)	0.506 (0.395)	>.91	0.076 (0.282)
Average baseline CD4	−0.764 (0.647)	−0.000 (0.900)	>144	−0.073 (0.225)
Proportion of patients initiating at WHO Stage 4	1,347.008* (0.077)	1.152* (0.060)	<.10	−0.058 (0.329)
Dummy = 1 if Hospital	−316.472** (0.001)	−0.311** (0.000)	= 1 if Hospital	−0.202** (0.007)
Months of ART service provision	−8.525+ (0.129)	−0.007+ (0.143)	>24	−0.145* (0.063)
Patient-years of ART per year of operation	−0.093 (0.326)	−0.000 (0.224)	>300	−0.191** (0.048)
			>800	−0.111+ (0.116)
Dummy = 1 if Rural			= 1 if Rural	−0.050 (0.580)
Dummy = 1 if Private			= 1 if Private	−0.039 (0.673)
Number of observations	45	45		45
R-squared	0.518	0.589		0.643
p of Early Start∧^c^	0.200	0.184		0.374
p of Economies of Scale	0.333	0.232		0.027

Note: **p<0.05,

* p<0.1,+p<0.2. p-values are given in parentheses to the right of the coefficients in columns (1), (2) and (4).

c Values of p for “Early Start” are the significance level at which the joint hypothesis can be rejected that neither baseline CD4 nor proportion of patients initiating at WHO stage IV influences the dependent variable.

### Sensitivity Analyses

In the discussion that follows, “cost-effectiveness” refers to results based on site-specific projections of future costs and benefits following 16 weeks of treatment.

#### One-way sensitivity analyses

Table 7 displays the cost-effectiveness of the ART program as the value of six key input variables are varied from 50% to 150% of their base cast values. Results are most sensitive to the estimated DALYs averted per death averted. This in turn, depends on disease progression rates, which are subject to measurement error and can vary from setting to setting. Although far less important, the death rate in the untreated comparison group and the cost of second-line ARV regimens are also important determinants of cost-effectiveness.

**Table 7:** One-way sensitivity analysis.

		Cost per DALY averted	
Model input	Base case value	50% of base case value	150% of base case value
Discounted DALYS averted per death averted	7.3	$1,665	$555
Deaths per 100 PY in HBAC comparison group			
• Baseline CD4∶000 - 050	116	$1,097	$754
• Baseline CD4∶050 - 199	27	$1,097	$754
• Baseline CD4∶200+	7	$1,097	$754
Weighted avg. cost of 2nd-line ARV regimen	$897	$680	$985
Weighted avg. cost of 1st-line ARV regimen	$259	$773	$892
Discount rate	3.0%	$885	$786
Ave. regimen switch rate (5 years of observation)^1^	2.2%	$799	$862

1 See Table 1 for switch rate per year per successive year of treatment.

Cost per DALY averted when six key model input variables are varied to 50% and 150% of base case values.

#### Multivariate sensitivity analysis

A Monte Carlo simulation assessed the aggregate uncertainty from the six variables shown in Table 7. In the absence of information about the distribution of input values, Beta distributions with minimum and maximum values set to 50% and 150% of the base-case value were fit around each variable of interest. The Alpha and Beta parameters were set to 3, ensuring a symmetrical distribution approximating the Normal with the base case as the mean value. [16] With 20,000 trials, the incremental cost-effectives varied between $642 and $1,152 at the 80% confidence level (SD = $205) and $570–$1,357 (SD = $205) at the 95% confidence level.

#### Scenario analyses

In CDC’s HBAC program in Uganda, 68% of all reduced DALYs were due to reduced AIDS mortality. The rest were attributed to reduced mortality in HIV uninfected children (21%); new adult HIV cases prevented due to lower plasma viral loads and behavior change (9%); and improved health status of patients who would not otherwise have died (2%). If one applied the same proportionate increase in DALYS for these benefits which were not measured in the CIDRZ program, cost per DALY averted in the analysis cohort would be $566 per DALY averted.

## Discussion

While in aggregate CIDRZ spent $556 per person-year delivering ART over the five years covered by these data, the average CIDRZ facility consumed $638 of resources per person-year of ART delivered. ART costs $833 per DALY averted when compared incrementally with CTX prophylaxis which conferred a 46% reduction in mortality over 1.5 years of follow-up in a trial conducted in Tororo District, Uganda. [17] If results are confined to the empirical data observed in this study the cost per DALY averted is $1,210. Two-thirds of all costs are consumed on-site, with the rest consumed in supervision, training, laboratories and logistics above the site. Bivariate analysis suggests that facilities with higher adherence rates spend more per patient, while facilities with more experience, greater scale or located in urban areas spend less.

Multivariate analysis of average total cost per patient-year and its two components, average on-site and average off-site costs, explains 78% of the variation in average total cost and 66% of the variation in average on-site cost. Maintaining the assumption that the facility incurs the extra cost of about $100 per patient-year for adherence greater than 91%, estimated average total cost per patient-year varies from a high of $1,025 to a low of $425. These estimates can be used by policy makers to gauge the likely impact of scale-up on total ART expenditure depending on whether they primarily expand treatment in the types of facilities that have higher or lower estimated costs. Furthermore, they suggest that some policies that would reduce on-site costs, such as out-sourcing to private providers or locating facilities in rural areas, would need additional funding in the form of off-setting increases in central support, which could negate the on-site cost savings.

Strengths of our study include its very large cohort size and the availability of detailed electronic clinical, pharmacy, laboratory and financial data to inform our cost-effectiveness estimates. The study is limited by the relatively short period of observed follow up, on average 1.4 years per patient. The ten-year time horizon incorporated into the model allows us to estimate the dynamics of costs and benefits as they change over time. The analysis is also by the lack of an exact comparison group of patients who were eligible for treatment but not receiving ART. Our reliance on patient data from Uganda for this purpose introduces uncertainty into our effectiveness estimates, but not our cost estimates. It thus is not expected to affect substantially our analysis of covariates associated with cost or cost-effectiveness.

Several published studies of the cost of ART have been completed on ART delivery sites in southern Africa. [18]–[27] In addition, there are five published cost-effectiveness studies on facility-based ART services,[28]–[32], one on home-based care in rural Uganda, [15] and one on the incremental cost-effectives of facility-based, mobile and home-base care in Uganda. [33] In addition a conference abstract was presented on the costs of scaled-up treatment in Malawi. [34].

Direct comparisons of these cost-effectiveness results cannot be made without substantial adjustments for the diversity of methods, outcome measures and the differing economic status of the study sites. For example, two of the cost-effectiveness analyses compared ART with no intervention, rather than with cotrimoxazole prophylaxis. One of these found that ART was cost-saving for patients with AIDS due to savings in hospitalization and other health expenditures, and cost $675 per life-year gained for non-AIDS patients. Another found that ART cost $1,631 per quality-adjusted life-year (QALY) gained for all treated HIV patients. [29], [30] A third study that ranked ART’s incremental cost-effectiveness against an array of other HIV prevention and treatment options found an incremental cost ranging from $547 to $5,175 (international dollars) per DALY averted. [28] A study using a simulation model to predict disease progression and treatment costs in a Cote d’Ivoire cohort found ART to cost $620 per life-year gained when compared with cotrimoxazole prophylaxis and an incremental $1,180 per life-year gained if the ART initiation decision incorporated CD4 test results. [31] The HBAC program in rural Uganda, which included a comprehensive valuation of ART’s prevention benefits, found cost-effectiveness of $597 per (age-weighted) DALY averted compared to cotrimoxazole alone. [15] Finally, the comparison of facility, home and mobile treatment services found that facility-based services had the most favorable cost-effectiveness at $1,396 per quality-adjusted life year (QALY) gained ($3,212 discounted 10-years costs/2.3 discounted QALYs). The Malawi cost study found very low unit costs ($237 per person-year of ART), owing to low antiretroviral drug costs, low personnel costs, and perhaps also to a carefully standardized treatment protocol. [35].

As documented in a recent review article of the costs of ART, unit costs are highly variable in both low and middle-income countries. [36] The median cost per patient-year in the six studies from low-income countries was $792. ARV drugs constituted 64% of the total or a median of $428 per person-year. This exceeds the average of $236 for ARV drugs per person-year found at the CIDRZ sites. Had CIDRZ had an ARV cost of $428, unit costs at the CIDRZ sites would have been $754. While the low costs at the CIDRZ sites can be explained by the difference in ARV drug costs, it is worth noting the wide observed variation in unit costs reported in this review, $682–$1,089 among the low-income countries studied, and $156–$3,904 among the lower middle-income countries. CIDRZ’s low ARV drug costs can be attributed in large measure to the use of the inexpensive stavudine-lamivudine- nevirapine combination for which it paid $156 per person-year of treatment. This regimen constituted 48.1% of all first-line regimens prescribed and 47.6% of all regimens dispensed during the study period. As the use of stavudine declines in accordance with WHO guidelines, and second-line therapies constitute a larger portion of prescriptions, CIDRZ ARV drug costs may rise. [37].

The diversity of methods, comparators, outcome measures and the differing economic status of the study sites make direct comparison of the cost-effectiveness of ART programs in sub-Saharan Africa difficult. However, most fall within a range of $500–$1,100 per life-year gained or DALY averted. The cost-effectiveness of the CIDRZ program is roughly in the middle of this range, and demonstrates that competitive cost-effectiveness can be maintained in a treatment program at scale. Our analysis also demonstrates that several characteristics of facilities and of their treatment policies have a large influence on average cost and its breakdown between on- and off-site components. While there may be room for cost-savings through the reduction of off-site costs, our results suggests that some strategies to reduce on-site costs will requiring commensurate and off-setting increases in off-site costs, which might negate any possible cost-savings.

## Supporting Information

Technical Appendix S1 **Additional detail on approach to the multivariate analysis; additional detail on** **Table 4** **.**
(DOCX)
