# Supplementary material for: Taking ART to Scale: Determinants of the Cost and Cost-Effectiveness of Antiretroviral Therapy in 45 Clinical Sites in Zambia
Source: PLoS One. 2012 Dec 20;7(12):e51993. doi: 10.1371/journal.pone.0051993 (PMC3527397; doi:10.1371/journal.pone.0051993)
Supplement: Technical Appendix S1 — Additional detail on approach to the multivariate analysis; additional detail on Table 4 . (DOCX) [file pone.0051993.s001.docx]

**Technical Appendix S1:**

**“Taking ART to Scale: Determinants of the Cost and Cost-Effectiveness of Antiretroviral Therapy in 45 Clinical Sites in Zambia”**

**Additional detail on approach to the Multivariate Analysis**

We dropped the percent pediatric and the median age from the analysis because they were not statistically significant in any specification that includes the other variables. We compared a linear specification of the cost function with continuous measures of most right-hand-side variables to a semilog specification with dichotomous measures of all right-hand-side variables. The log transformation of the average cost dependent variables renders their distributions more symmetric and thus in closer conformity with the Gaussian distributional assumption required for regression’s desirable asymptotic properties. The availability of two definitionally distinct cost measures on each of the 45 facilities, off-site and on-site average costs, gives us two dependent variables to be explained by the same vector of independent variables and thus justifies our use of Zellner’s “seemingly unrelated regression” approach to improve the efficiency of parameter estimation and hypothesis tests. We used STATA version 12.0’s “*regress*” and “*sureg*” commands to implement these methods (STATA, College Station, TX, 2012).

**Additional detail on Table 4**

Table 4 presents the bivariate association between each of the eight predictors and three measures of efficiency: average total cost, average on-site cost and average on-site cost. Column (1) gives the variable names. For each continuous variable, we have also selected a threshold, which is given in column (2). The means and standard deviations of these variables and of their dummy variable representations are in columns (3) and (4). Column (5) gives the correlation coefficient between each of the continuous variables and average total cost. Columns (6) and (7) give the conditional means of average total cost for each value of the dummy variable. Column (8) gives the p-value of the association between the variable in that row and average total cost. For the dichotomous variables, the p-value is the result of a two-tailed t-test for the equivalence of the means under unequal variance. For the continuous variables, the p-value measures the statistical significance of the correlation coefficient in column (5) which measures the linear association of that row variable with average total cost. This pattern is repeated for average on-site costs in columns (9) through 12 and for average off-site costs in columns (13) through (16).
